# Editorial: Novel Approaches for Studying Creativity in Problem-Solving and Artistic Performance

**DOI:** 10.3389/fpsyg.2019.02059

**Published:** 2019-09-18

**Authors:** Philip A. Fine, Amory H. Danek, Kathryn J. Friedlander, Ian Hocking, William Forde Thompson

**Affiliations:** ^1^School of Psychology and Wellbeing, University of Buckingham, Buckingham, United Kingdom; ^2^Department of Psychology, Universität Heidelberg, Heidelberg, Germany; ^3^School of Psychology, Politics and Sociology, Christchurch Canterbury University, Canterbury, United Kingdom; ^4^Department of Psychology, Macquarie University, Sydney, NSW, Australia

**Keywords:** creativity, problem solving, artistic performance, methodology, novel approach

## Introduction

Creativity can be observed across multiple domains of human behavior including problem solving, artistic and athletic engagement, scientific reasoning, decision making, business and marketing, leadership styles, and social interactions. It has a long history of research in many disciplines, and involves a variety of conceptual and methodological approaches. However, given its multi-faceted character, and the multidisciplinary (though not necessarily interdisciplinary) nature of creativity research, it is perhaps unsurprising that such research has tended to examine discrete areas of study, thereby adopting a focused approach that lacks opportunity for cross-fertilization. It is therefore important to encourage interdisciplinary discourse and novel methodological approaches to investigating all aspects of creativity. This can best be achieved by sharing and integrating research ideas, methods, and findings across multiple domains and disciplines, including but not restricted to psychology, neuroscience, philosophy, linguistics, medicine, education, and performance science.

The aim of this Research Topic is to showcase recent creativity research involving new methodological approaches across a range of creativity domains and academic disciplines. Broadly speaking, we see three ways by which such novel methodological approaches can develop. Firstly, adopting technologies such as brain stimulation and EEG allow researchers to investigate creativity in new ways, and new digital research platforms allow researchers to more easily access domain-specific online populations. Secondly, traditional methodologies, already shown to be effective in one field of creativity research, can be employed to investigate hitherto neglected creativity domains. Thirdly, taking advantage of the interdisciplinary nature of creativity research, we can interrogate one domain of creative performance using research perspectives from another, such as viewing medicine as a performance science akin to music (Kneebone, 2016) or investigating insight moments with magic tricks (Danek et al., 2014). This novel juxtaposition of methods from multiple domains and disciplines allows new research questions to be addressed. These three ways of developing novel methodological approaches thus involve: the development of novel methods; the novel application of tried-and-tested methods; and the novel combination of previously separate methodologies.

The Research Topic contains 27 articles (20 Original Research articles, one Case Report, one Review, and five methodological or theoretical contributions). Twelve address questions of creative cognition, covering insight, divergent thinking, and problem solving. Eleven articles investigate creative arts and artistic performance, with a further four addressing other aspects of creativity. Given the focus of the Research Topic, we have decided to address the articles in terms of their methodological approaches, rather than the type of creativity under investigation. Indeed, we hope to encourage the development and ultimately the wider application of those methodological approaches described herein to any aspect or domain of creativity.

## Tracking the Process: Physiological Approaches

In line with the increasing pace of technological advancement, several articles utilize physiological techniques to measure and manipulate the creative process, including the electroencephalogram (EEG), and transcranial current stimulation, both direct (tDCS) and alternating (tACS). Dolan et al. employ EEG in both music performers and selected audience members during prepared and improvised renditions of the same piece of classical music, demonstrating what they call an “improvisatory state of mind.” Truelove-Hill et al. measure resting-state EEG in their investigation of the effects of near-future and far-future priming on insight and analytical problem-solving. Di Bernardi Luft et al. use both EEG and tACS in their case study of a professional visual artist with exceptionally vivid spontaneous visual imagery during meditation sessions. They demonstrate increased occipital gamma oscillations during visual imagery, and an effect of alpha tACS on the contents of the artist's images. In another study of musical creativity, Anic et al. investigate the effects of both excitatory and inhibitory tDCS over the left hemisphere primary motor cortex (M1) of pianists who were improvising with their right hands: improvisations under excitatory tDCS were rated as significantly more creative, demonstrating the role of M1 in musical creativity.

Various other articles employ process-tracing methods to probe the creative process. Carey et al. investigate dance in a novel way, using pupillometry (a metric of mental effort) to demonstrate greater pupil dilation in novice, rather than intermediate, dancers as they performed or imagined dance movements. Jankowska et al. use both eye-tracking and think-aloud (verbal protocol) analyses whilst adults completed a creative drawing task, demonstrating methodological synergy between both types of process-tracing and various psychometric measures of drawing creativity. Spiridonov et al., Loesche et al., and Dolan et al. all track physical movement during various creative acts. Spiridonov et al. examine the classic 9-dot problem by tracking the position and movement of the solver's index finger on a tablet, and demonstrate specific patterns of motor behavior characterizing the differences between unsuccessful and successful solvers. Similarly, Loesche et al. investigate the chronology of insight moments in a novel insight eliciting task, “Dira,” by tracking the position of the mouse cursor, allowing them to better pinpoint the moment when solutions emerge. Finally, Dolan et al. investigate musical creativity in ensemble playing in various ways, including continuous 3D tracking of the musicians' movement. This enables them to explore movement pattern differences between improvised and prepared renditions, as well as demonstrate, for instance, that the flutist and pianist correlated their fast movements significantly more in an improvised rendition than a classically prepared one.

## The Time-Course of Creativity

One common theme, found in 10 articles, is the study of temporal or chronometric aspects of the creative and associated processes. Three articles involving process-tracing, focusing particularly on moment-to-moment aspects of the creative process, have already been mentioned (Loesche et al., Spiridonov et al., and Dolan et al.). Hass and Beatty directly compare performance on the Alternative Uses Task (AUT) and Consequences Task, showing that both approximate well to an exponential cumulative response time model; they also provide an explanation for why later responses are generally rated as more creative than earlier ones, known as the serial order effect. Kizilirmak et al. measure feelings of warmth (FoW) ratings for Compound Remote Associate Tasks as a function of task difficulty, whether it was successfully solved, and whether the solution (if it occurred) was an example of insight; they demonstrate that FoW ratings increase more abruptly for trials solved with compared to without an insight experience. Kupers et al. measure moment-to-moment ratings of novelty and appropriateness in their study of children's creativity using a novel coding framework. Botella et al. explore the stages of the creative artistic process, which they propose differs from both the creative process and the artistic process, by interviewing visual graphic arts students, integrating their findings into Creative process Report Diaries.

Rather than focusing on the creative process itself, three articles measure the time-course of associated processes. Wang et al. explore the temporal structure of semantic associations in an association chain task and its relationship to divergent thinking. Korovkin et al. use a dual-task procedure to track the temporal dynamics of working memory involvement throughout both insight and non-insight problem-solving experiences. Truelove-Hill et al. investigate the effects of a priming procedure on creative problem-solving by asking problem-solvers to think about the near vs. distant future in order to differentially impact their cognitive style, in accordance with construal level theory. They then apply growth-curve analysis in a novel way to uncover the time-course of these transient priming effects.

## Promoting and Measuring Creativity: Psychometric Approaches

Several articles describe novel approaches to promote, track or measure creativity. Three articles propose novel methods for inducing insight. Friedlander and Fine posit a new protocol for eliciting insight moments, that of cryptic crossword solving, drawing parallels between certain cryptic clue mechanisms and problem types already found in the insight literature, such as rebus puzzles, remote associate problems, anagrams, and jokes. Such an approach could be instrumental in exploring individual differences in insight ability, and identifying insight experts. In order to investigate multiple instances of both positive (Aha!) and negative (Uh-oh!) insight experiences, Hill and Kemp use the well-known adversarial game of Connect 4, asking participants to label each move as insight or search (either positive or negative) and collecting concomitant phenomenological ratings. Loesche et al. have developed a new game, “Dira,” based on the existing game “Dixit,” in which participants must find a connection between a short sentence and one of six visual images. However, only the image (or text) over which the mouse is hovering is clearly visible: this allows real-time process-tracing via mouse movements, and provides information about relevant metacognitive and behavioral mechanisms, such as the intensity of the insight moment.

Other cognitive methods applied to creativity research in the current articles include: the use of verbal protocol analysis to probe metacognitive and self-regulation mechanisms together with eye-movement measures during a creative drawing task (Jankovska et al.); the measurement of feelings of warmth during insight and non-insight puzzle solving (Kizilirmak et al.); and the application of the classic dual-task paradigm to investigate the effect of working memory load on solving insight and non-insight problems (Korovkin et al.). Camic et al. also describe the potential utility, for those with dementia, of Visual Thinking Strategies (VTS), an arts-based facilitated learning methodology involving moderated group discussions, permitting individuals to create meaning through viewing visual art.

Two articles probe novel and interesting causal relationships between creativity and other cognitive activities or processes. Having a broad attentional scope has previously been shown to enhance creativity, but Wronska et al. demonstrate the reverse relationship, that divergent thinking can broaden visual attention on a subsequent visual scanning task and enhance peripheral target recognition. Osowiecka and Kolanczyk show that silently reading poetry can both increase and decrease divergent thinking performance, depending on the type of poetic metaphors, the poetic narration style, and individual differences in long-term exposure to poetry.

Several articles explore novel psychometric methods for measuring and otherwise quantifying aspects of creativity. Threadgold et al. present a newly validated normative pool of 84 rebus puzzles freely available for future use in problem-solving and insight studies. Kupers et al. propose a micro-level domain-general systematic coding framework for measuring novelty and appropriateness of creative products on a continual basis. Kershaw et al. apply a novel originality scoring method, the Decision Tree for Originality Assessment in Design (DTOAD), to creative ideation within engineering design. Clements et al. adapt Amabile's Consensual Assessment Technique (CAT; Amabile, 1982; Cseh and Jeffries, 2019) for online use so as to have a broader reach, by which they investigate the effects of varying levels of dance expertise and experience on ratings of choreographic creativity. Loesche et al.'s exploration of the chronometry of insight moments and Threadgold et al.'s construction of a normative database of rebus puzzles both treat the strength of the Eureka experience as a continuum rather than a dichotomous all-or-none phenomenon, which has generally been a more common approach; similarly, some articles, including Hill and Kemp, and Loesche et al., consider phenomenological correlates of the insight moment as continua.

## Technological and Methodological Advances

In addition to the studies using tDCS, tACS, and EEG already mentioned, two articles in particular employ methods novel to creativity research to increase the reach of their studies. For their direct comparison of the AUT and the Consequences Task, Hass and Beatty's participants were recruited from Amazon Mechanical Turk (MTurk) using psiTurk, an open-access web-app which interfaces with MTurk, allowing online experimental control and response collection. In their study of choreographic creativity, Clements et al. use an online version of the CAT together with a snowball sampling technique in which participants could rate as few or as many as they wished out of 23 randomly ordered short videos: this yielded 2153 individual ratings from 850 raters.

Camic et al. advocate the use of wearable technology for measuring psychophysiological changes on a continuous basis during creative behaviors, particularly where it is important that such data collection is unobtrusive, for instance in persons with dementia. Wearable technology such as wristbands can record 3D position using accelerometers, as well as physiological indices of arousal and stress including heart rate, heart rate variability, skin conductance, and skin temperature. Finally, in their Perspective article, Gobet and Sala advocate the use of methods in Artificial Intelligence (AI), which they argue are less susceptible to mental set issues, in both the design of new experiments and the generation of new theory in relation to the study of creativity.

## Investigating Creative People and Populations

Several articles focus more on the creative person, by studying either specific (and sometimes less-studied) populations, or interpersonal aspects of teamwork, ensemble, and co-creativity. Hogan et al. investigate budding fashion designers on a reality television programme in which they are tasked with designing garments. The authors analyze the designers' thinking dispositions using qualitative analysis of the programme transcripts in terms of the 8 Studio Habits of Mind. In a multi-institutional wide-ranging Conceptual Analysis article, Camic et al. explore how we can conceptualize and understand artistic creativity in the dementias, a population easily and undeservedly overlooked in creativity studies. An interesting aspect of the article is their discussion of co-creativity, which focuses on shared processes. Hocking, too, addresses co-creativity, in his dyadic case study of the subjective experience of a professional artist as seen through the eyes of a psychological researcher and thus artistic collaborator, using Interpretative Phenomenological Analysis (IPA). Another case study of an artist (Di Bernardi Luft et al.) employs neuroimaging to investigate spontaneous vivid visual imagery, central to this artist's creativity. Though still focusing on the creative process, Kupers et al. present two case studies specifically investigating children's creativity, exemplified by two empirical examples, a music composition task and the solving of a physics problem: their coding framework will no doubt also be applicable to adults (and to other domains of creativity).

Other articles addressed questions of interpersonal interaction with reference to teamwork and ensemble. Reiter-Palmon and Murugavel demonstrate the utility of problem construction in teams by studying the social and cognitive processes involved. Both Bishop and Dolan et al. investigate aspects of ensemble playing and collaborative processes in music performance. Bishop reviews recent literature on collaborative musical creativity, in terms of how ensembles achieve creative spontaneity, through the lenses of embodied music cognition, emergence, and group flow. Dolan et al. explore synchrony of movement in ensemble music performers as a function of the level of improvisation.

## Multidisciplinary, Interdisciplinary, and Blended Methodological Approaches

As noted in the introduction to this editorial, one of the main drivers of this Research Topic is that of fostering interdisciplinary cross-fertilization. Two articles explicitly use such a multidisciplinary approach. Wang et al. combine approaches from computational linguistics, complex systems, and creativity research in their investigation of the relationship between semantic association and divergent thinking tasks. Camic et al.'s article about artistic creativity in the dementias is the culmination of a 2-year interdisciplinary study involving research psychologists and neurologists, artists, and media professionals.

Certain articles, although focusing more on a single discipline (often psychology), use a blended approach of multiple methods, some comparing different methodologies directly, such as Hass and Beatty's comparison of the AUT and the Consequences Task. Dolan et al., in their study of an improvisatory approach to performing classical music, measure various performance-related parameters, post-performance ratings from both performers and audience members, EEG signals again from both performers and selected audience, and 3D motion tracking of the performers' movements. This broad range of measures enables them to demonstrate convergent evidence for differences between improvised and prepared musical performances. Jankowska et al. integrate psychometric, eye-tracking, and verbal protocol analysis in their study of creative drawing. Finally, Carey et al. combine measures of motor imagery, dance performance, and pupillometry to investigate dancers' learning of dance moves.

## The Future of Creativity Research

Given the breadth of creativity research, investigating as it does at least the creator, the creative process, the creative product, and environmental influences on creativity (Rhodes, 1961; Abdulla and Cramond, 2017), it is important to integrate research ideas, methods, and findings across diverse disciplines. The 27 articles in this Research Topic present a broad picture of contemporary creativity research across multiple disciplines and domains. Separately and together they present a range of novel approaches for studying all aspects of creativity which we hope will encourage further interdisciplinary cross-fertilization. Creativity research is clearly thriving, and through the methodological creativity of developing innovative research methods and approaches, we are in a strong position to advance our understanding of creativity in all its forms.

## Author Contributions

PF wrote the first draft of this editorial, and all authors equally contributed to the revisions.

### Conflict of Interest Statement

The authors declare that the research was conducted in the absence of any commercial or financial relationships that could be construed as a potential conflict of interest.
